# Polymeric Nanoparticles and Chitosan Gel Loading Ketorolac Tromethamine to Alleviate Pain Associated with Condyloma Acuminata during the Pre- and Post-Ablation

**DOI:** 10.3390/pharmaceutics13111784

**Published:** 2021-10-25

**Authors:** Salima El Moussaoui, Ismael Abo-Horan, Lyda Halbaut, Cristina Alonso, Lluïsa Coderch, María Luisa Garduño-Ramírez, Beatriz Clares, José Luis Soriano, Ana Cristina Calpena, Francisco Fernández-Campos, Mireia Mallandrich

**Affiliations:** 1 Department of Pharmacy, Pharmaceutical Technology and Physical-Chemistry, Faculty of Pharmacy and Food Sciences, University of Barcelona, Av. Joan XXIII 27-31, 08028 Barcelona, Spain; selmouel9@alumnes.ub.edu (S.E.M.); iabohora7@alumnes.ub.edu (I.A.-H.); halbaut@ub.edu (L.H.); anacalpena@ub.edu (A.C.C.); mireia.mallandrich@ub.edu (M.M.); 2 Institute of Advanced Chemistry of Catalonia-CSIC (IQAC-CSIC), 18-26 Jordi Girona St., 08034 Barcelona, Spain; cristina.alonso@iqac.csic.es (C.A.); luisa.coderch@iqac.csic.es (L.C.); 3 Centro de Investigaciones Químicas, Universidad Autónoma del Estado de Morelos, Avenida Universidad 1001, Cuernavaca 62209, Mexico; lgarduno@uaem.mx; 4 Department of Pharmacy and Pharmaceutical Technology, School of Pharmacy, University of Granada, 18071 Granada, Spain; jlsoriano@correo.ugr.es; 5 Institut de Nanociència i Nanotecnologia IN2UB, Universitat de Barcelona, 08028 Barcelona, Spain; 6 Reig-Jofre Laboratories, Av. de les Flors s/n, 08970 Sant Joan Despí, Spain; ffernandez@reigjofre.com

**Keywords:** PLGA nanoparticles, chitosan gel, Condyloma acuminata, ketorolac tromethamine, anti-inflammatory, topical delivery

## Abstract

This study describes the preparation and evaluation of two formulations, a hydrogel and a nanostructured system, containing ketorolac tromethamine as an anti-inflammatory agent for the local therapy against the inflammatory process derived from the surgical excision of Condyloma acuminata. Both formulations were physicochemically characterized. In vitro release profiles show that the nanoparticles release 92% ± 2.3 of the total ketorolac tromethamine encapsulated, while the chitosan gel releases 18.6% ± 0.2. The ex vivo permeation and distribution through human skin were also assayed and was observed how the main amount of ketorolac tromethamine is retained in the epidermis. In vivo studies were accomplished to evaluate the anti-inflammatory efficacy in mice which also involved the histological analysis to confirm the in vivo results. The nanoparticles present a significantly higher anti-inflammatory efficacy than chitosan gel. The tolerability of developed formulations was assessed by monitoring the biomechanical properties of the skin before and after application of both formulations. No statistical differences in trans-epidermal water loss and skin hydration with respect to the basal values were observed and the formulations exhibited higher anti-inflammatory activity compared to a reference ketotorlac tromethamine solution. Therefore, it can be concluded that both formulations can be proposed as outstanding candidates for offering a local anti-inflammatory therapeutical tool with potential clinical application.

## 1. Introduction

Sexually transmitted infections (STIs) represent nowadays a significant public health problem [1]. One of the most common STIs is the human papillomavirus (HPV) infection [2], being Condyloma acuminata (CA) its clinical manifestation [3]. To date, more than 200 HPV strains have been sequenced, of which 40 are known to cause genital infections [2]. There are two types of HPV in terms of the oncogenic risk, of which the virus subtypes 6 and 11, are classified as of low oncogenic risk, and are those which are mainly responsible 90% of cases. It is noteworthy that subtypes 16 and 18 in contrast are deemed as high-risk strains, due to their oncogenic potential [4]. Patients aged 20 to 39 account for more than 70% of CA infections [5]. Children [6] and pregnant women can also be affected [7].

Even though asymptomatic Condyloma acuminata is common, certain cases might be accompanied by itching, pain, and bleeding [8]. The treatment strategy could be ablative or by using topical procedures based on the localization, size and morphology of the lesions [2]. In some cases, such as intra-anal involvement, the ablative strategy might be the only option [5]. However, Condyloma acuminate is known for its recurrence, particularly if HPV 11 has been diagnosed [9]. In previous studies, our research group investigated the use of topical ketorolac tromethamine (KT) as a good alternative for managing the inflammatory process following the excision of anogenital warts with promising results by loading the drug in alginate-based hydrogel [4]. KT is a non-steroidal anti-inflammatory drug (NSAID) belonging to the Aryl acetic acid derivatives. It is used for the relief of postoperative pain and the management of inflammation [10]. 

Stemming from the nanoparticles (NPs) having the property of sustained drug release, the drug can reach the skin over a prolonged time period and can be retained in the skin at the desirable concentration [11]. In this context poly (D, L-lactic-co-glycolic acid) (PLGA) has demonstrated immense potential among the matrixes used for NPs owing to its biocompatible and biodegradable properties, among others [12].

Chitosan (CTS), a natural polymer, is a promising candidate for controlled drug delivery systems and wound healing [13]. It being a strong candidate is due to their properties such as biocompatibility, biodegradability, antimicrobial activity, low immunogenicity, mucoadhesivity, hydrophilicity and ease modification [14]. Additionally, CTS can also provide antifungal properties and hemostatic properties as well as the anti-inflammatory effect [13,14]. All these properties are of great interest for the management and care of the areas treated post-surgically, thus positively influencing the reduction of complications such as bleeding and infections and this favors the wound healing process [15].

Skin is the largest human organ which accounts for roughly 10% of an average total body mass. The main function of skin is to act as a barrier preventing moisture and the loss of nutrients and protecting against ultraviolet (UV) radiation action, chemicals, allergens and microorganisms [16]. Transdermal Drug Delivery (TDD) offers various benefits over other traditional drug delivery routes such as it is painless and non-invasive with fewer side effects, and more patient compliance among other benefits [17]. *Stratum corneum* (SC) is the outer layer of the epidermis with a crucial barrier function. Many strategies have been investigated to overcome this barrier and facilitate the drug permeation [18,19]. However, overcoming the SC to reach the deeper layers of skin or the systemic circulation is not always appropriate. In certain cases, such as Condyloma acuminata, where the infection occurs in the epidermis, taking advantage of the drug accumulation in this skin layer would be a great strategy for local treatment. 

Taking all the above into account, in this work, we formulated KT into a PLGA nanostructured system as a vehicle for preventing the inflammatory process during the surgical excision of warts. The KT-NPs may be easily sprayable onto warts without the need to contact them. At the same time, we formulated KT into a CTS-based hydrogel as a vehicle for managing the local inflammatory process stemming from the post-operative stage in the surgical excision of warts taking advantage of the mucoadhesive and regenerative properties of CTS. Both formulations were characterized and evaluated in vitro, ex vivo and in vivo.

## 2. Materials and Methods

### 2.1. Materials

KT, polyvinyl alcohol (PVA) with 90% hydrolyzation, ethyl acetate (EA), lactic acid solution 85%, phosphate buffer solution (PBS) pH 7.6, gentamicin sulphate and bovine serum albumin were purchased from Sigma-Aldrich (Barcelona, Spain). CTS was obtained from FagronIberica (Terrassa, Spain). PLGA (Resomer^®^ RG 503) was acquired from Boehringer Ingelheim (Ingelheim, Germany). Adhesive tapes were obtained from D-squame, (Cuderm Co., Dallas, TX, USA). Double distiller water was obtained from a Milli-Q1 Gradinet A10 system apparatus (Millipore Iberica S.A.U., Madrid, Spain). All the other chemicals and reagents used in the study were all of analytical grade (Panreac, Castellar del Vallès, Spain).

### 2.2. Preparation of Formulations

#### 2.2.1. Preparation of CTS Gel

CTS gel was prepared weighting an accurate amount of CTS and dispersing the polymer in 1% lactic acid solution under continuous stirring so as to obtain a final concentration of 3% (*w/v*) until dissolution. Then, the mixture was allowed to swell in the fridge overnight. Appropriate amount of KT (2%; *w/v*) was added once the gel was swollen and gently homogenized. Table S1 in Supplementary Materials depicts the critical process parameters and Table S2 shows the Quality Target Product Profiles.

#### 2.2.2. Preparation of NPs

NPs were prepared using the double emulsion-solvent evaporation method as described previously with modification [20,21]. The NPs were optimized by a two-level full factorial design for 3 factors in standard order, which led to 8 candidate formulations (Table S3). In brief, 250 µL of the inner aqueous phase pH 2.0 (W_1_) consisting of a PVA 2% (*w/v*) and KT (80 mg/mL) solution were added to 1 mL of the oily phase (O), which contained 90 mg of PLGA in ethyl acetate. A probe Vibra-Cell^®^ sonicator (Sonics and Materials, Inc., Newtown, CT, USA) was used to homogenize this mixture for 20 s in two 10 s cycles at 50 watts and 30% amplitude, resulting in a primary emulsion (W_1_/O), in which 2 mL of external aqueous phase (W_2_), PVA 2% (*w/v*), was added drop-wise and subjected to a second sonication process for 1.5 min, 10 s cycles (50 watts and 30% amplitude) to produce the double emulsion (W_1_/O/W_2_). Finally, a volume of 5 mL of PVA 0.3% (*w/v*) was added to the double emulsion before the organic solvent was evaporated under reduced pressure (Bücchi B-480, Flawil, Switzerland) for about 15 min at 36 °C and 74 mbar vacuum. For comparison studies, blank NPs were also fabricated. Table S1 shows the critical process parameters and Table S2 shows the Quality Target Product Profiles.

### 2.3. Physical Characterization of Formulations

#### 2.3.1. Characterization of CTS Gel

##### pH Study

The pH was recorded in triplicate by pH meter micropH 2000 (Crison Instruments SA, Alella, Spain) at room temperature.

##### Swelling and Degradation Tests

The swelling ratio (SR) was assayed based on a gravimetric method. For this task, known amounts of dried hydrogel were immersed in PBS (pH = 5.5) for 3 h at 32 °C. Samples were removed and weighed (W_t_) after blotting the surface water at pre-established times. The PBS uptake was measured in triplicate. The swelling ratio was determined using the following equation:

(1) $$\mathrm{SR}=\frac{W_{s}-W_{d}}{W_{d}}$$

where W_s_ is the weight of the swollen hydrogel at various times and W_d_ is the weight of dried hydrogel.

The gel degradation rate was evaluated in terms of percentage of weight loss (WL). Briefly, known amounts of dried hydrogel were immersed in PBS (pH = 5.5) at 32 °C for 3.75 h. Samples (*n* = 3) were then dried and weighed at pre-established time intervals. The following equation was used to calculate the WL:

(2) $$\mathrm{WL}\left(\%\right)=\frac{W_{i}-W_{d}}{W_{i}}100\%$$

where W_i_ denotes the initial weight of the hydrogel and W_d_ denotes the weight of the hydrogel at various times.

Both, the swelling and the degradation were fitted to mathematical models. The best fit was chosen based on the determination coefficient value (R^2^).

##### Morphological Studies

Scanning Electron Microscopy (SEM) was used to visualize the internal structure of developed hydrogel in a JEOL J-7100F (JEOL Inc., Peabody, MA, USA). Samples were carbon-coated with an Emitech K950X coater (Quorum Technologies Ltd., Kent, UK).

##### Rheological Study

The rheological properties of CTS gel were analyzed in a rotational HaakeRheo Stress 1 rheometer (Thermo Fisher Scientific, Karlsruhe, Germany) with cone-plate geometry (Haake C60/2° Ti, 60 mm diameter, 0.105 mm cone-plate gap). The shear profile was evaluated following a ramp-up stage (0–100 s^−1^ for 3 min), a constant shear rate stage (100 s^−1^ for 1 min), and a ramp-down stage (100–0 s^−1^ for 3 min). All measurements were taken in triplicate at room temperature and the steady-state viscosity was recorded at 100 s^−1^ (constant shear stretch). 

Experimental results were then fitted to different mathematical models by regression analysis [4].

#### 2.3.2. Characterization of the NPs

##### Physicochemical Characterization

Both, the mean particle size (Zave), the polydispersity index (PI) and the zeta potential (ZP) of the KT-NPs were determined by dynamic light scattering (DLS). The characterization was carried outby diluting the samples 1/20 in Milli-Q water and measuring by a Zetasizer Nano Zs (Malvern Instruments, Malvern, UK) at 25 °C with disposable cuvettes.

The pH of the resulting colloidal suspension of NPs was measured by pH metermicrop H 2000 (Crison Instruments SA, Alella, Spain).

Transmission electron microscopy (TEM) technology was used to evaluate the structure of KT-NPs with a JEOL 1010 (JEOL Inc., Tokyo, Japan) at 80 Kv. Uranyl acetate (1% *w/v*) was used as a staining solution to visualize the NPs. The image processing program Image J (1.53 e) was utilized to measure the particle size starting from the produced TEM image.

##### Encapsulation Efficiency

An indirect method was used to determine the KT encapsulated in the NPs based on the measuring of the non-entrapped KT in the dispersion medium. For this purpose, fresh KT-NPs were centrifuged for 15 min at 14,000 rpm (Sigma 301K, Sigma Laborzentrifugen GmbH, Osterode am Harz, Germany). Subsequently, free KT was separated using Ultracel YM-100 filters (Millipore, Bedford, MA, USA) and collected in the filtered solution. 

The determination of the KT was performed using a high-performance liquid chromatography (HPLC) methodology which had been previously validated. The encapsulation efficiency (EE) was calculated using Equation (3).

(3) $$\mathrm{EE}\%=\frac{\mathrm{Total}\;\mathrm{amount}\;\mathrm{of}\;\mathrm{KT}-\mathrm{free}\;\mathrm{KT}}{\mathrm{Total}\;\mathrm{amount}\;\mathrm{of}\;\mathrm{KT}}$$

### 2.4. Stability Studies

The physical stability of KT-NPs stored at 4 °C was studied for 3 months by monitoring potential changes in Zave, PI and EE. 

The stability of the KT-CTS gel was visually evaluated. The formulation was stored at room temperature for 3 months. 

### 2.5. In Vitro Drug Release Study

To investigate the KT release from NPs and the CTS gel, vertical Franz diffusion cells (Vidrafoc, Barcelona, Spain) with a diffusion area of 2.54 cm^2^ were used. A dialysis membrane (MWCO 12,000–14,000 Da., Medicell International Ltd., London, UK) was placed between the donor and receptor chambers in the case of NPs and nitrocellulose 0.45 µm pore size (Millipore Merck, Madrid, Spain) for CTS gel. The receptor medium was PBS solution pH 7.4. The system was kept at 32 ± 0.5 °C under continuous stirring, maintaining sink conditions throughout the course of the experiment. 200 µL of KT-NPs (*n* = 6) and 200 mg of CTS gel (*n* = 6) were put into the donor compartment. Aliquots of 300 µL were removed at predetermined intervals of up to 9 h and replaced with an equivalent volume of tempered receptor medium. The experimental data were fitted to first order, using the Prism software, v. 5 (GraphPad Software, Inc., San Diego, CA, USA).

### 2.6. Ex Vivo Permeation Studies

The penetration and permeation of KT in and through skin was evaluated ex vivo using human skin. The skin was obtained from abdominoplasties (protocol code N°001 approved on 20/01/2016 by Bioethics Committee of the Barcelona-SCIAS Hospital); which was frozen and kept at −20 ± 5 °C until the date of the permeation test. Then, specimens were dermatomed at 500 µm pieces. The human skin pieces were allowed to thaw at room temperature and then were placed between the donor and receptor chambers of Franz static diffusion cells (Lara-Spiral, Courtenon, France). The Franz cells consisted of a 3 mL receptor volume and 1.86 cm^2^ diffusion area.

The experimental system used PBS (pH 7.6), gentamicin sulphate 0.04% (*w/v*) to prevent skin degradation [22] and bovine serum albumin 1% (*w/v*) as receptor fluid at 32 °C continuously stirred at 500 rpm by a magnetic bar allowing sink conditions throughout the test. 10 μL samples (either NPs or CTS gel) were placed on the donor chamber.

At the end of the experiment (24 h), skin membranes were demounted, and the remaining formulation amounts were recovered by a swab. Equally, the receptor fluid was collected to measure the amount of KT distributed at each skin layer [9]. KT was extracted from the skin samples by sonication in a water:methanol (1:1, *v/v*) medium for 20 min. KT was determined by HPLC. The extracted amount of KT is expressed as μg/cm^2^ and percentage (%) of the applied dose. Control cells were also used to evaluate potential interferences in the sample analysis of the receptor fluid or skin samples using unloaded gel and blank NPs.

### 2.7. HPLC Analysis

KT in samples was determined using HPLC methodology validated in terms of linearity, accuracy, and precision according to ICH Q2 (R1) validation guidelines. The HPLC system consisted of a Waters 717 plus Autosampler with a detector Waters 2487 (Waters, Milford, MA, USA); a C18 column YMC-Pack Pro, 250 × 4.6 mm, 5 µm (YMC Co., LTD, Kyoto, Japan) was used with a flow rate of 1 mL/min at isocratic conditions. The mobile phase was carried out with acidified water (glacial acetic acid 1.65%) and acetonitrile with 0.065% of triethylamine at the elution conditions of 1:1 *v/v*. The volume of injection was 10 μL. The wavelength was set at 314 nm.

### 2.8. In Vivo Anti-Inflammatory Efficacy Evaluation

#### 2.8.1. TPA-Induced Ear Oedema in Mice

Male Swiss CD-1 mice (20–25 g) purchased from Circulo AND, S.A. de C.V. (Coyacan D.F., Mexico) were kept under standard animal housing conditions in a 12 h dark-light cycle with access to food and water ad libitum. This study was approved by the animal research ethical committee of the Vivarium at University of Morelos (protocol code BIO-UAEM 03:2019 date of approval February 2019). 

The protocol was carried out as previously reported by Domínguez-Villegas et al. [23]. A 12- O-detradecanoylphorbol-13-acetate (TPA) solution of 50 mg/mL in ethanol was used to induce mouse ear oedema inflammation. Animals were divided into three groups of three specimens each one (*n* = 3). 5 µL of TPA solution was applied to both sides of the right ear simultaneously with 100 mg of formulation (KT-CTS gel to group 1 and KT-NPs to group 2) as well as 5 µL of ethanol to both sides of the left ear. The control group was treated with an equal amount of KT solubilized in acetone applied to both sides of the right ear and 5 µL of acetone to both sides of the left ear. The animals were then sacrificed by cervical dislocation 4 h after treatment and 7 mm circular sections were cut from the left and right ears. These were weighted accurately. The anti-inflammatory activity expressed as a percentage of inhibition of the inflammatory process was calculated according to the Equation (4):

(4) $$\mathrm{Inhibition}\left(\%\right)\;=\;\left(\frac{\mathrm{Weight}\;\mathrm{control}\;\mathrm{ear}-\mathrm{Weight}\;\mathrm{treated}\;\mathrm{ear}}{\mathrm{Weight}\;\mathrm{control}\;\mathrm{ear}}\right)\;\times \;100$$

#### 2.8.2. Histological Analysis

Histological analysis of the circular sections was conducted after TPA-induced oedema in mice to evaluate the anti-inflammatory efficacy by observing whether immunologic cells were present in the tissues. Hence, the circular ear sections were fixed in 4% formaldehyde solution and then embedded in paraffin to obtain histological cuts which were stained with hematoxylin-eosin and observed by light microscopy. 

### 2.9. In Vivo Tolerance Study

The study was carried out with 10 healthy-skinned female participants ranging in age from 21 to 64 years old. The study was authorized by the University of Barcelona’s Ethics Committee (IRB00003099, approved on 20 March 2018), which followed the Declaration of Helsinki’s guidelines. All the participants provided written informed consent forms. Volunteers were asked to not use cosmetic or another skin care products on the test areas during two days before the study. The subjects stayed in the test room for at least 30 min before taking the measurements. Several readings were collected from the flexor side of the left forearm before applying the formulation (baseline readings). Then, readings were collected newly just after application of a uniform layer (0.1 mL/cm^2^) of formulation at1 h after application and at 2 h. The total quantity of water lost (TEWL) through the skin was measured using a Tewameter^®^ TM 300. (Courage-Khazaka electronic GmbH, Cologne, Germany). The hydration of the stratum corneum (SCH) was determined using a Corneometer^®^ CM 825. (Courage-Khazaka electronic GmbH, Cologne, Germany). All measurements were taken in accordance with published procedures.

### 2.10. Statistical Analysis

All statistical analyses were performed using the Prism^®^ software, v. 5 (GraphPad Software Inc., San Diego, CA, USA). T-student tests were performed on the in vitro release and the ex vivo permeation studies to compare both formulations. Furthermore, analysis of variance (ANOVA) was conducted on the in vivo anti-inflammatory efficacy study to compare the formulations, as well as in the in vivo tolerance study to compare the biomechanical properties if the skin over time to the basal values. The level of statistical significance was set at *p* < 0.05.

## 3. Results

### 3.1. Characterization of Formulations

#### 3.1.1. Characterization of CTS Gel

The resulting CTS gel was transparent and extremely viscous with poor flowability. The pH of the CTS gel was 4.4 ± 0.1 and 5.5 ± 0.1 for KT-CTS gel. 

**Figure 1:**
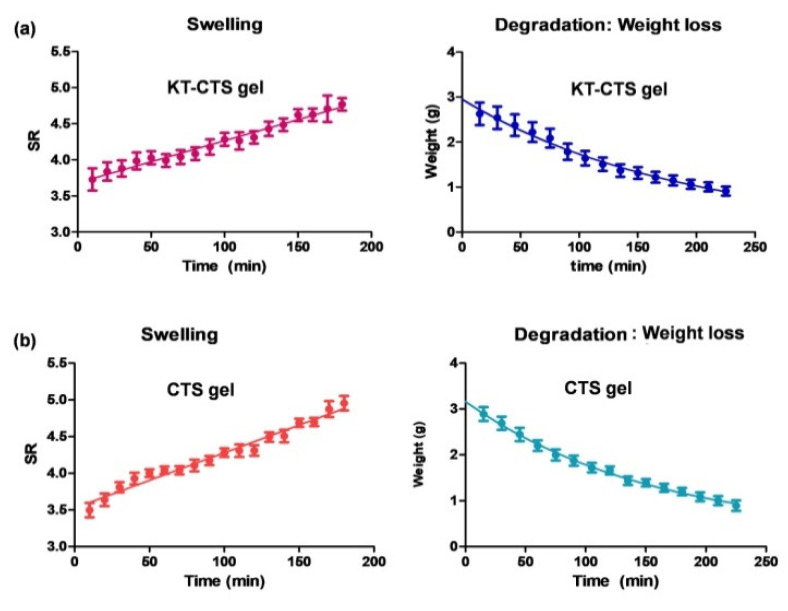
Swelling and degradation processes of KT-CTS gel. Modelling graphic expressed as mean ± SD (*n* = 3). Panel (**a**) shows the KT-CTS gel; and panel (**b**) the blank gel without KT (CTS gel).

Figure 1 shows the swelling and degradation profiles of KT-CTS gel and blank CTS gel. Similar profiles were observed for both gels, indicating that the addition of KT in the gel does not affect the gel structure. Nevertheless, significant statistical differences were found between the KT-CTS gel and blank CTS gel in terms of the initial weight on the swelling study. It can be seen that the swelling is a linear and steady process following a zero-order kinetic with a lag-time of 10 min. This formulation was able to absorb PBS up to 4.5-fold its weight.

After 3.75 h ± 0.22 h, the CTS gel had utterly degraded in the medium (PBS). The weight loss fitted to a one-phase exponential decay. Table 1 shows the fitted parameters values for both processes.

**Figure 2:**
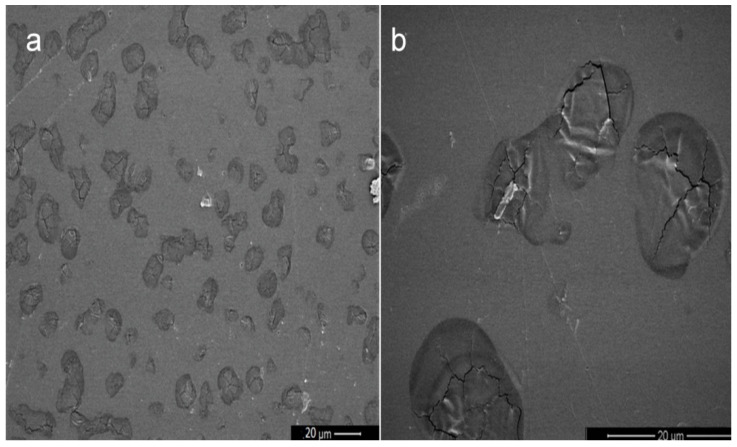
SEM images of the dried discs of KT-CTS gel. (**a**) Magnification 1000×; and (**b**) magnification 5000×; the scale bar is 20 µm length.

The surface of the dried KT-CTS gel was evaluated by SEM. The gel presented a compact and dense appearance, with smooth surface which presented micro-irregularities where the water and KT may be interposed within the polymer structure (Figure 2).

**Figure 3:**
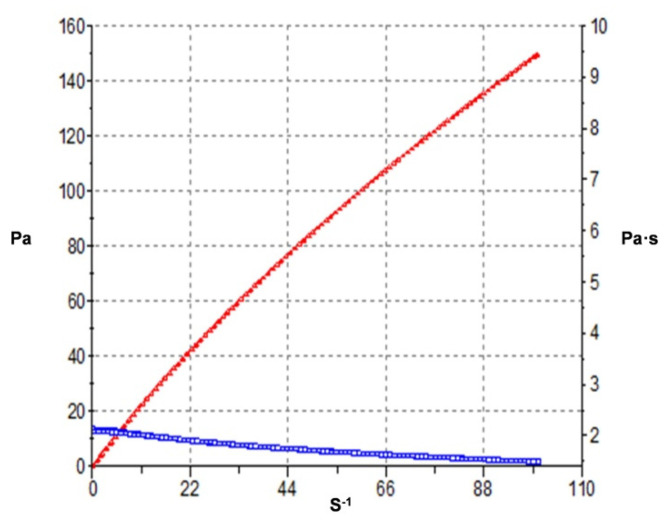
Rheological profile of KT-CTS gel. Blue curve represents the formulation viscosity. Red curve represents the shear stress of the formulation.

To predict the potential topical usage of KT-CTS gel, the rheological study was carried out. The mathematical model that best fitted experimental flow data was the Casson model (r^2^ = 0.999), confirming a shear-thinning behavior of KT-CTS gel. In Figure 3 is depicted the rheological profile which revealed that the gel has a pseudoplastic behavior with a viscosity of 1.431 Pas ± 0.0004. 

#### 3.1.2. Characterization of NPs

The resulting KT-NPs showed a high EE, concretely 93.91%. The Zave was 108.9 ± 2.3 nm with a PI value of 0.061 indicative of narrow distribution, and a ZP of −6.20 mV. The pH of the external aqueous phase W_2_ resulted in 5.2.

**Figure 4:**
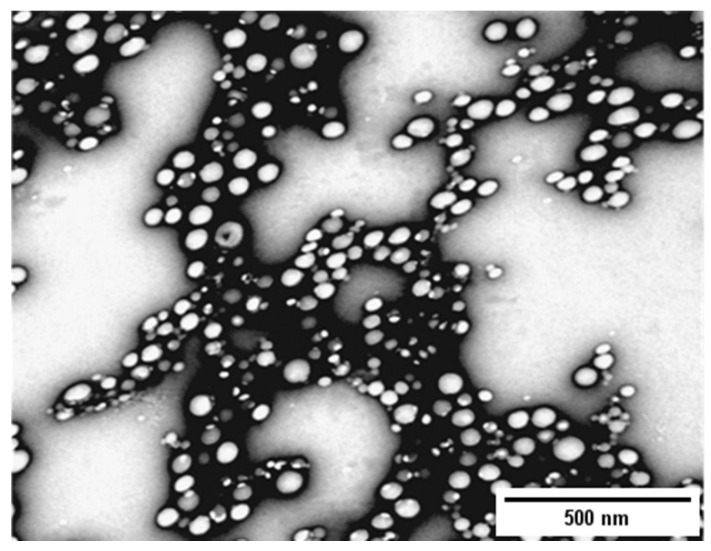
TEM image of KT-NPs.

As depicted in Figure 4, the TEM image shows round-shaped structure of KT-NPs. The particle size associated was 51.9 ± 14.5 nm (26.4 nm was the minimum size, whereas the maximum was 92.7 nm). 

### 3.2. Stability Studies 

The stability of the formulations was evaluated at 3 months, stored at 25 °C and 4 °C for the KT-CTS gel and the KT-NPs, respectively. After 3 months of storage of KT-CTS gel at room temperature (25 °C), no visual changes in appearance were evidenced.

The KT-NPs stored at 4 °C for 3 months neither showed statistically significant changes in the Zave, the PI, the ZP nor the EE (Table 2). The statistical significance level was set at *p* < 0.05.

### 3.3. In Vitro Drug Release Study

**Figure 5:**
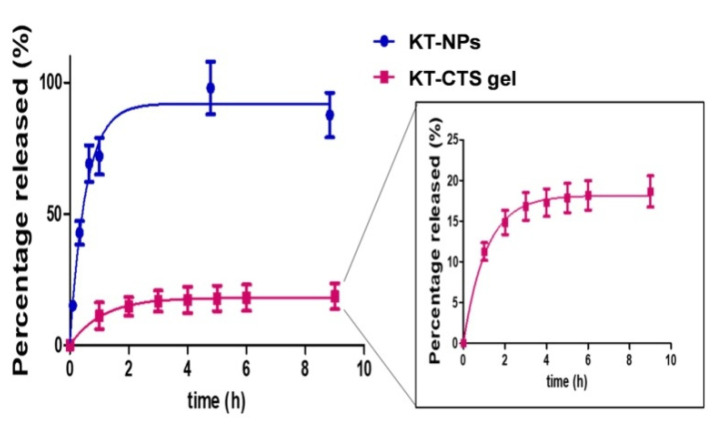
In vitro release profile of the formulations, KT-NPs and KT-CTS gel (*p* < 0.001). Each value represents mean ± SD (*n* = 6).

The release profiles of both formulations were investigated by the in vitro diffusion cells technique. The NPs allowed a higher degree of release of KT than the CTS gel (Figure 5). 

According to the drug release study, the best-fitted model to the experimental data was the first-order kinetic equation with a K value of 0.901 h^−1^ for KT-CTS gel and 1.841 h^−1^ for the KT-NPs (Table 3). Significant statistical differences were found for all the parameters evaluated.

### 3.4. Ex Vivo Permeation of KT through Human Skin

Ex vivo permeation studies were carried out to examine the permeability of KT through human skin and investigate the biodistribution of KT in the skin layers. A large percentage of KT was found on the skin surface in the residual formulation, for both, the NPs and the CTS gel as depicted in Table 4. NPs provided greater absorption of KT than the CTS gel. In the case of NPs, the KT was mainly retained in the epidermis whereas it was in the receptor fluid in the case of the CTS gel. The CTS gel led to higher percutaneous absorption of KT than the NPs. However, no significant statistical differences were found in the KT amount retained or in the epidermis or in the amount percutaneously absorbed. Statistical differences were only observed in the amount diffused into the receptor fluid and the amount penetrated in the *stratum corneum*.

### 3.5. In Vivo Anti-Inflammatory Efficacy Evaluation

#### 3.5.1. TPA-Induced Ear Oedema in Mice

**Figure 6:**
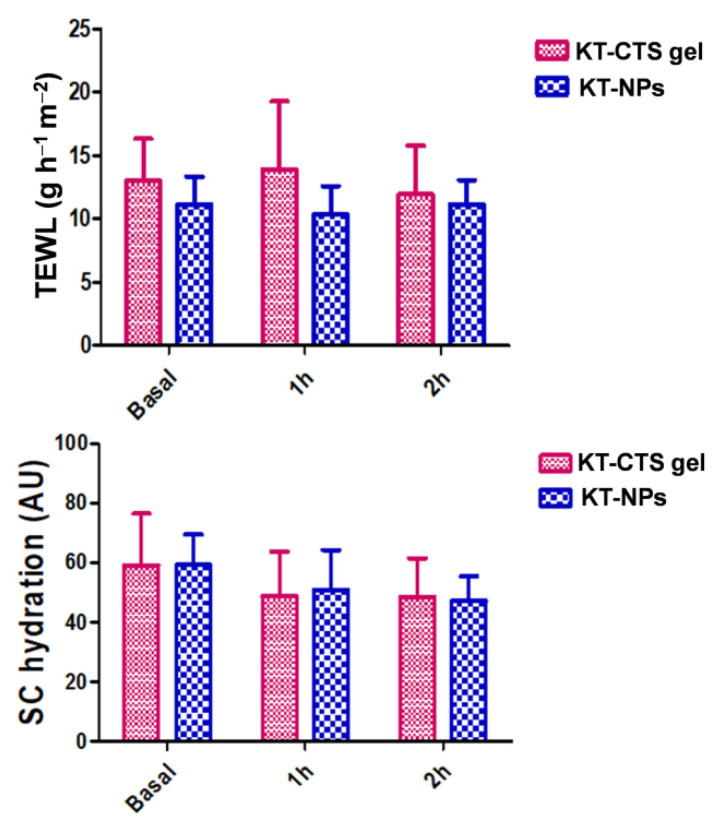
Biomechanical properties in human volunteers before the formulations applications (Basal) 1 h and 2 h post-application: TEWL for both formulations; and the evolution of the stratum corneum hydration for both formulations.

The acute ear skin oedema model created by topical administration of TPA was used to assess the formulations anti-inflammatory activity. As a reference of inflammatory inhibition, a KT solution in acetone was used. Figure 6 shows the percentage of inhibition for each formulation. 

The formulation of the NPs was the one that exhibited the highest reduction in inflammation, and both formulations showed greater anti-inflammatory efficacy than the reference KT solution in acetone. All differences were statistically significant.

#### 3.5.2. Histological Analysis

**Figure 7:**
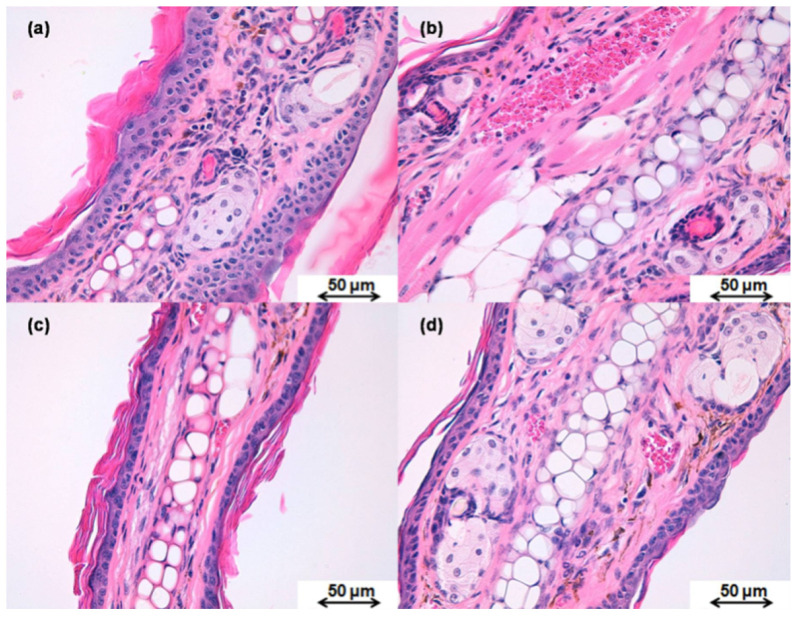
Histological images of the mice ear after the TPA-induced ear oedema in mice. (**a**) negative control ear; (**b**) positive control ear; (**c**) ear treated with KT-NPs, and (**d**) ear treated with KT-CTS gel. The scale bar is 50 µm in length. The small purple cells corresponding to the immune system cells.

After the TPA-induced inflammation, a histological evaluation of the mice ears was conducted Figure 7a,b shows the histological images for the negative and positive controls, respectively, as well as for the treated tissues with either KT-NPs (Figure 7c) or KT-CTS gel (Figure 7d). No immune system cells were found in the negative control ear (Figure 7a). On the contrary, the positive control shows immune system cells in blood vessels (Figure 7b). The ear treated with KT-NPs did not present immune system cells (Figure 7c). A limited number of immune cells systems were observed in the blood vessels of the ear treated with KT-CTS gel (Figure 7d).

### 3.6. In Vivo Tolerance Study

**Figure 8:**
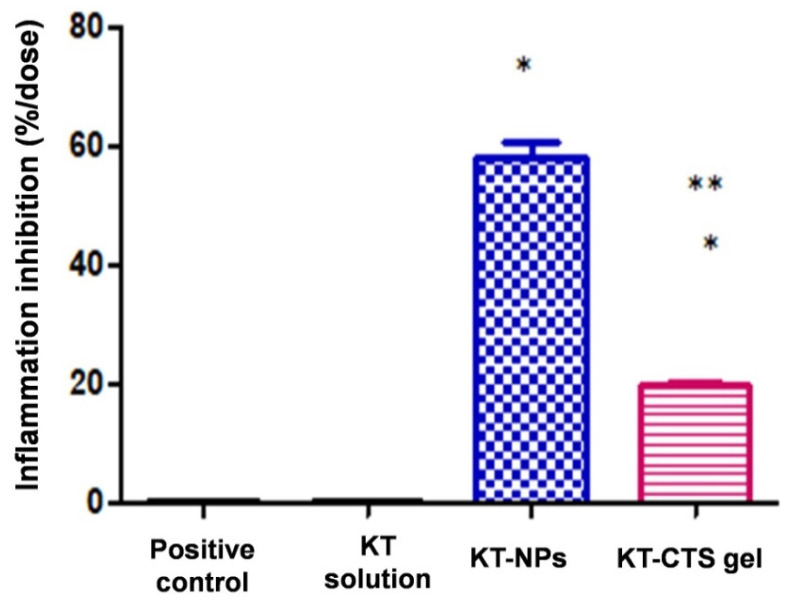
Anti-inflammatory efficacy of studied formulations expressed as a percentage reduction in in-flammation when compared to a control. The results are presented as the mean ± SD of three measurements. * stands for statistical differences between the formulations and the positive control and the KT solution, ** stands for statistical differences between KT-NPs and KT-CTS gel (*p* < 0.0001).

Figure 8 shows the evolution of biomechanical parameters as tracked before and after the formulations were applied for up to 2 h. This was to assess the impact of the formulations on the skin hydration and its integrity. TEWL is measured in grams per hour per square meter (g/h·m^2^) and the *stratum corneum* hydration is given in arbitrary units (AU). A slight decrease was observed in the *stratum corneum* after the application of the formulations whereas no changes in the TEWL values were observed. Neither were anystatistical differences found for TEWL and nor were they found in the process of *stratum corneum* hydration at 1 and 2 h compared to the basal values. 

## 4. Discussion

The patients’ health-related quality of life is negatively impacted by Condyloma acuminata and therefore finding appropriate and effective treatments is necessary. Improving this quality of life can be achieved by alleviating the pain associated with the disease, an aim which has been pursued with considerable effort. In this context, the current study is focused on developing two formulations containing KT as an anti-inflammatory agent to address this need, one for the pre-operative period and another for the post-operative period. KT was selected based on its promising results previously reported by our group [4], into which it was formulated into a topical sodium alginate-hydrogel and tested on human skin and porcine vaginal mucosa. This showed its capacity to be released from the formulation and to permeate through the tissues, and it also exhibited an optimal anti-inflammatory activity. Two carriers were chosen based on extensive research previously reported: polymeric NPs and CTS hydrogel. Several studies have shown the potential of KT in the management of postoperative pain and the impact of using it as a pre-operative agent and that it added towards achieving better outcomes during and after surgery [24,25,26]. The hypothesis was that the KT-NPs might be an appropriate choice in the pre-operative period as CTS might be used as a spray and facilitate their clinical applications whereas the KT-CTS gel would be beneficial in the post-operative period, due to the biodegradable, mucoadhesive, homeostatic and antibacterial properties of CTS, among its other properties.

Hydrogels are three-dimensional structure composed of hydrophilic polymeric networks, capable of absorbing large amounts of water or biological fluids. The polymers are water-insoluble due to the presence of chemical (covalent bonds) or physical crosslinks (non-covalent secondary interactions such as Van der Waals, hydrophobic, hydrogen bonding and electrostatic interactions). These crosslinks provide the structure of the network and its physical integrity. The hydrogels have thermodynamic compatibility with water, which allows them to swell in an aqueous medium [27,28]. The morphological characteristics of the KT-CTS gel showed that it has a compact and dense structure. This was also observed by Yanget al. [29]. Nevertheless, other researchers observed lamellar or porous structures in CTS gels [30,31,32,33]. The differences in the microstructure observed by the different authors may be attributed to the formulation or preparation of the sample. 

Hydrogels can also show a swelling behavior that depends on the characteristics of the external environment and as a consequence the polymer complexes can break down, swell or show drastic changes in their swelling values as a result of changes in certain physiological stimuli. Some of the factors that influence the polymers that respond to certain physiological stimuli include pH, temperature, ionic strength, and electromagnetic radiation [34]. In our case, the KT-CTS gel swelling process followed a zero-order kinetic. This can be interpreted as being due to the excellent ability of CTS to absorb large volumes of water [33], a property which opens up the possibilities for drug delivery and wound healing applications [35]. Our findings indicate that the dehydrated gel is hygroscopic absorbing to an extent of up to 4.5 times its weight in water in less than 4 h. Several researchers have investigated the swelling phenomenon of polyionic or polyelectrolytic hydrogels. In some cases, the swelling balance and the mesh size were caused by the percentage of ionic component and by the degree of ionization of the active principle [36]. For example, Peniche et al. [28] verified the ability to complex salicylic acid and acrylic acid derivatives with cationic polyvalent ligands such as CTS. On the other hand, Gabrielii et al. [37] studied the swelling properties of CTS hydrogels under the influence of various pH and sodium chloride concentrations (0.075 M and 0.15 M). The authors deduced that the effect of adding sodium chloride could be explained by the increase in the total amount of ions that decreases the ratio between the ions inside the hydrogel and the external environment. Therefore, under extreme pH conditions (pH = 3 and pH = 12) the degree of swelling decreases when the ionic strength of the medium increases. Our findings demonstrate that the CTS gel diffuses into the media, indicating that it should be expected that this formulation degrades successfully when submerged in a biological medium.

The hydrogel viscosity plays a crucial role in controlling drug release and skin penetration [23]. The Casson rheological model describes the viscoelastic fluids flow [38]. This behavior supports the intended topical application of this formulation, thus facilitating its outpatient application. On key point in favor of it is that it be applied in the desired area precisely, without fear that it will disperse to other regions as soon as the patient changes postural position, and on the other hand, it can flow and its viscosity decrease when stress is applied, which is a desirable feature in the potential spread of dermatological products as it allows a smooth and painless application.

The preparation of NPs by the double emulsion method requires ultrasound or high-speed homogenization, followed by evaporation of the solvent either by continuous magnetic stirring at room temperature or under vacuum conditions. Generally, the polymer is dissolved in an organic solvent that is used in the oil phase, while the aqueous phase contains the stabilizer [39]. First, the hydrophilic drug and the chosen surfactant are dissolved in water and, a solution of the polymer is dissolved in an organic solvent. The primary emulsion is prepared by dispersing the aqueous phase in the organic phase. Afterwards, a re-emulsification is carried out with a second aqueous phase that also uses surfactant. Finally, the solvent is evaporated and the NPs are collected. Evaporation of the solvent can be carried outby magnetic stirring at room temperature for several hours or by rotary evaporation [39,40]. NPs production has many independent variables. The polymer used to formulate the NPs affects the structure, properties and applications of the particles, so there is no single polymer suitable for all drugs. The PLGA was chosen to design the NPs because of its various advantageous reported characteristics, especially biodegradability [41]. There are two characteristics that need to be considered when formulating polymeric NPs for a given route of administration: particle size and encapsulation efficiency. For example, if fast absorption is intended, the nanoparticle size should be 100 nm or below. The ZP is a measure of the particle charge, the greater the absolute value of ZP, the greater the charge on the NPs surface, and in turn, the greater stability of the particles [40]. In the case of KT-NPs the small ZP value was not critical because the KT-NPs were prepared with PVA as the stabilizer agent. The PVA mechanism of action to prevent particle aggregation relies on steric hindrance [42]. The obtained KT-NPs showed a high encapsulation efficiency, round-shaped structure and narrow distribution which indicates a monodisperse system. The difference in the value of Zave between TEM and DLS measurements is attributed to the fact that the DLS measured the hydrodynamic diameter, whereas the TEM shows the real particle size [20]. Zambaux et al. [43] studied the influence of certain experimental parameters (preparation temperature, solvent evaporation method, surfactant concentration, polymer molecular weight) on the Zave, PI and the ZP of NPs prepared by the double emulsion method. The authors concluded that high concentrations of surfactant (3% *w/v*, or higher) guaranteed a successful emulsification process, and smaller particles which came with a satisfactory PI. Other factors influence the characteristics of PLGA NPs prepared by the double emulsion method W/O/W solvent evaporation. Bilati et al. [21] studied the influence of the sonication process, analyzing the modification of the size and distribution of NPs as a function of the duration and intensity of sonication. The work showed that the duration of the second homogenization step, the one that provides the double emulsion (W/O/W), had a greater influence on the particle size than the first homogenization (W/O emulsion). The particle size decreases with the increasing sonication time of the second emulsification. The results of the study also suggested that the intensity of sonication influenced the morphometry of the NPs as well. Additionally, the organic solvent used can also influence the final properties of NPs. Mainardes et al. [44] evaluated this effect in his work, comparing the particle size obtained when preparing the PLGA NPs with two organic solvents: methylene chloride or ethyl acetate. It was concluded that methylene chloride provided larger NPs than when ethyl acetate was used as organic solvent. The selection of the method depends, therefore, on several factors that must be evaluated in order to obtain the NPs with the desired characteristics and properties [40].

The pH of the external aqueous phase of the KT-NPs as well as of the KT-CTS gel were within the pH range of the healthy skin so the formulations are not expected to cause any disorder [45,46].

Whether a hydrogel is suitable as an active ingredient delivery system in a certain area depends to a large extent on its polymeric structure. The controlled release of active principles from polyionic hydrogels can respond to changes in environmental parameters, such as: pH, temperature and other stimuli. Thus, interpolymeric hydrogels such as polyacrylic acid/polyvinyl alcohol or polymethacrylic acid linked to polyethylene glycol can form reversible complexes depending on the temperature and pH conditions of the external environment [36,47]. Our findings revealed that the KT release from the NPs was significantly faster than CTS gel. A more outstanding level of release was observed from NPs in comparison to CTS gel (*p* < 0.0001). The KT-NPs release profile seems to be fitting with the hypothesis of our study as the KT fast release is crucial for the pre-operative application where a fast release is preferred. Meanwhile, the profile of KT-CTS gel, which could be due to the electrostatic interaction between KT and CTS, and the compact structure, seems to be suitable for the post-operative application, where a more sustained release is desirable. 

Hydrogels have also been used as vehicles for active ingredients that can interact with the mucosa of the gastrointestinal tract, the colon, the vagina, the nasal mucosa, and other parts of the body thanks to their ability to prolong their times of residence in the body place of administration. In the present work, we have assessed the biodistribution of KT after the skin was exposed to both formulations under a finite dose approach for 24 h. To determine the amount of KT in each compartment, the skin was separated into its different layers, and both the receptor fluid and the residual formulation at the end of the experiment were recovered for further drug quantification. We observed that the greatest amount of KT remained on the skin surface for both formulations. This is in line with previous hydrogels works, and it is because of the primary barrier function of the *stratum corneum* against the drug penetration [4,48]. Both formulations seem to be adequate vehicles for the dermal delivery. In both cases, KT readily diffuses through the skin, and it is mainly retained in the epidermis, where it can exert its anti-inflammatory and analgesic properties. Similar profiles were observed for KT hydrogels formulated with different polymers [4]. When the distribution of KT into the human skin was tested with an alginate-based hydrogel, the diffusion of KT was limited to the epidermis where similar amounts of KT were found with poor retention of KT in the dermis and not quantifiable KT in the receptor fluid. Nor was KT retained in the dermis from a Carbopol-based and Pluronic-based hydrogels, and there was also poor diffusion of KT into the receptor fluid. Among these polymers, CTS gel is the vehicle which most enhanced the diffusion of KT into the receptor fluid. 

In order to evaluate the potential changes in the skin characteristics caused by the application of the formulations, the variations in the properties of the skin were studied using bio-metrological techniques. The parameters evaluated were the *stratum corneum* hydration and the TEWL. These parameters are indicative of a potential irritant or pro-inflammatory effect. TEWL measurement, due to passive diffusion of water through the skin, provides important information on skin barrier function and its integrity [49]. An increase in TEWL values reflects a possible deterioration in the barrier function, leading to decreased protection against water loss. The TEWL values 2 h after post-application of the formulations, showed slight variation with no significant statistical differences compared to the basal values. In healthy skin functioning normally as a barrier, TEWL should be directly proportional to skin hydration [50]. *Stratum corneum* hydration is determined as the conductance that free water provides to the skin surface [51]. Even though the *stratum corneum* hydration experienced a decrease after the application of the formulations, the differences observed were not found to be statistically significant. Our results show that the skin exhibit sufficiently hydrated values (above 45 AU), according to the skin hydration normal values in standard conditions [4,52]. In the case of the KT-CTS gel, the decrease can be probably related to the CTS gel swelling behavior when it is applied to the skin. 

The anti-inflammatory efficacy study revealed that the KT-NPs exhibit statistically significantly higher anti-inflammatory efficacy than KT-CTS gel by an approximately three-fold ratio. These results agree with the histological analysis which showed the presence of the immune cells system in the positive control, in agreement with TPA-induced oedema. Thanks to the KT-NPs action, the infiltration of immune system cells is completely inhibited, while the ear treated with the KT-CTS gel histology shows a limited number of these cells in tissue blood vessels. Even so, the KT-CTS gel anti-inflammatory efficacy is evident, as shown in the histological images. It should be noted that the NPs loaded less KT than the KT-CTS gel. This demonstrates how NPs are an excellent formulation for the application of KT since we achieved a powerful effect without the need to apply a high dose, thus improving the side effects window and, therefore, patient satisfaction and adherence, for pain during treatment is one of the factors that affects treatment adherence [53,54,55].

Considering that the formulations show suitable characteristics for the topical delivery, are well-tolerated and exhibited a good anti-inflammatory profile, both formulations are candidates for offering a local anti-inflammatory activity in the clinical application. In this regard, future pre-clinical and clinical studies might be conducted.

## 5. Conclusions

Two polymeric formulations loading KT were developed to address the pain associated with the ablation of anogenital warts in Condyloma acuminata. The polymers were chosen based on their biocompatibility and biodegradability among other characteristics. The CTS was selected because of its muco-adhesivity, and hemostatic and antimicrobial activity, while the PLGA was selected because it is already approved by several medicine regulatory authorities.

The formulations, a CTS gel and a polymeric nanostructured system, were fully characterized. Both formulations exhibited suitable features for the dermal application and remained stable for at least 3 months. They showed a fast drug release, particularly the KT-NPs, and both formulations led to the accumulation of KT in the epidermis where the drug can exert its anti-inflammatory effect. Even though the NPs were prepared at a lower concentration of KT than the CTS gel, they showed greater retention of KT in the epidermis than the CTS gel.

Both formulations showed efficacy in reducing the inflammation in TPA-induced ear oedema in mice and both formulations were well-tolerated in healthy human volunteers.

The faster release, the greater amount of KT in the epidermis and the superior anti-inflammatory efficacy of the NPs over the KT-CTS gel led us to consider the KT-NPs as a good formulation in the pre-operative period, meanwhile, the viscosity and rheological profile of the KT-CTS gel in combination with the known mucoadhesive and antimicrobial properties of the CTS may confer some advantages for the post-operative period in the surgical removal of anogenital warts.

## Figures and Tables

**Table 1:** Best-fit values of the swelling and degradation processes of CTS gel with and without KT.

	Parameter	Swelling	Degradation
KT-CTS gel	Y_0_ (g) ^1^	3.681 ± 0.050 *	2.952 ± 0.168
	K ^2^	0.006 ± 0.001	0.005 ± 0.002
	R^2^	0.9033	0.9322
CTS gel (blank)	Y_0_ (g) ^1^	3.526 ± 0.062	3.164 ± 0.183
	K ^2^	0.008 ± 0.001	0.006 ± 0.002
	R^2^	0.9085	0.9341

^1^ Y_0_ = initial weight; ^2^ K = degradation constant (g/min) or swelling constant (min^−1^); * statistical differences between the KT-CTS gel and blank CTS gel (significance level set at *p* < 0.05).

**Table 2:** Comparison of the particle size (Zave), polydispersity index (PI), zeta potential (ZP), pH of the external aqueous phase and encapsulation efficiency (EE) from the KT-NPs at time 0 and time 90 days (*n* = 3) stored at 4 °C. Results are expressed as mean ± standard deviation.

Parameter	KT-NPs Day 0	KT-NPs Day 90	*p*-Value
Zave (nm)	108.9 ± 2.3	111.2 ± 3.6	*p* = 0.4039
PI	0.061 ± 0.013	0.064 ± 0.018	*p* = 0.8265
ZP	−6.20 ± 0.48	−6.28 ± 0.39	*p* = 0.8337
pH aqueous external phase (W_2_)	5.2 ± 0.1	5.2 ± 0.2	*p* = 1.000
EE%	93.9 ± 2.83	92.4 ± 2.45	*p* = 0.5258

**Table 3:** Parameters of KT release from KT-CTS gel and KT-NPs according to first-order kinetics. Values are expressed as the Mean ± standard deviation (*n* = 6).

Parameter	KT-CTS Gel	KT-NPs	*p*-Value
%R∞ (%) ^1^	18.6 ± 0.3	92.0 ± 2.3	*p* < 0.0001
K (h^−1^) ^2^	0.9 ± 0.2	1.8 ± 0.1	*p* < 0.0001
Half-time (h)	0.8	0.4	*p* = 0.0014
R^2^	0.9422	0.9287	-

^1^ %R∞ represents the maximum percentage of KT released; ^2^ K represents the release rate.

**Table 4:** Skin distribution of KT (expressed as μg/cm^2^) contained in the formulations with an in vitro test after an exposure time of 24 h. Values are expressed as mean ± SD (*n* = 6).

Biodistribution	KT-NPs (µg/cm^2^)	KT-CTS Gel (µg/cm^2^)	*p*-Value
Total applied	14.61	26.91	-
Skin surface	12.14 ± 1.31	26.51 ± 0.84	-
Stratum corneum	0.08 ± 0.02	0.04 ± 0.01	0.03 *
Epidermis	0.71 ± 0.32	0.32 ± 0.17	0.13
Dermis	0.001 ± 0.001	0.002 ± 0.001	0.57
Receptor Fluid	0.02 ± 0.01	0.49 ± 0.09	<0.01 *
Total recovery	12.95 ± 0.84	27.36 ± 0.85	-
Percutaneous Absorption	0.73 ± 0.32	0.81 ± 0.19	0.71

* Statistical differences between formulations (*p* < 0.05).

## Data Availability

The data presented in this study are available on request from the corresponding author. The data are not publicly available due to they are part of a Doctoral Thesis and it will be available once the Thesis will be published.

## Supplementary Materials

The following are available online at https://www.mdpi.com/article/10.3390/pharmaceutics13111784/s1, Table S1: Critical Process Parameters (CPPs) and Critical Quality Attributes (CQAs) for the KT-formulations, Table S2: Quality Target Product Profiles (QTPPs) for the KT-formulations, Table S3: Optimization of the KT-NPs by a Two-level Full Factorial Design for 3 factors in standard order, and Table S4: Responses from KT-NPs formulated based on the Two-level Full Factorial Design for 3 factors.
