# Supplementary material for: Polymeric Nanoparticles and Chitosan Gel Loading Ketorolac Tromethamine to Alleviate Pain Associated with Condyloma Acuminata during the Pre- and Post-Ablation
Source: Pharmaceutics. 2021 Oct 25;13(11):1784. doi: 10.3390/pharmaceutics13111784 (PMC8618351; doi:10.3390/pharmaceutics13111784)
Supplement: Supplementary file 1 [file pharmaceutics-13-01784-s001.zip › pharmaceutics-1383843-supplementary (1).pdf]

pharmaceutics

MDPI

Supplementary Materials: Polymeric Nanoparticles and Chitosan Gel Loading Ketorolac Tromethamine to Alleviate Pain Associated with Condyloma Acuminata during the Pre- and Post-Ablation

Salima El Moussaoui, Ismael Abo-Horan, Lyda Halbaut, Cristina Alonso, Lluïsa Coderch, María Luisa Garduño-Ramírez, Beatriz Clares, José Luis Soriano, Ana Cristina Calpena Francisco Fernández-Campos and Mireia Mallandrich

Table S1. Critical Process Parameters (CPPs) and Critical Quality Attributes (CQAs) for the KT-formulations.

	Critical Process Parameters (CPPs)	Specification	Critical Quality Attributes (CQAs)	Specification (value and range)
**KT-CTS gel**			pH of the final formulation	Eudermic (4.0 – 6.0)
			Swelling ratio (SR)	SR = 4.7 after 3h (4.0 – 5.4)
	pH		Degradation rate	In 3.75h (3.10 – 4.40) CTS gel degraded in the medium (PBS).
	Order of raw materials addition	As described in section 2.2.1		
	CTS concentration	3% (*w*/*v*)	Rheology behavior (viscosity)	Pseudoplastic behavior 1.431 Pas (1.420 – 1.440)
	mixing time and speed (homogenization)	Avoid stirring at high speed so as not to form air bubbles in the hydrogel. ca. 50 rpm 5 min.	Microscopy Morphology	compact and dense, with smooth surface.
			Release ability	K = 0.9h $^{-1}$ (0.3 – 1.2); %R∞ = 18.6% (16.8 – 20.4)
			Permeation capacity (skin retention)	Superficial skin layers, manly in the epidermis
**KT-NPs**			pH aqueous external phase (W_2)	5.2(4.0 – 6.0)
	Order of addition of raw materials	As described in section 2.2.2	particle size (Zave)	108.9 nm (95.1 – 122.7)
	Sonicator power and cycle length	W_1/O: 50 watts 30% amplitude for 20s in 10s cycles	polydispersity index (PI)	0.061 (≤ 0.1)
			efficiency (EE%)	93.9% (87.0 – 102.0)

Organic solvent and its evaporation method	$W_1/O/W_2$: 50 watts 30% amplitude for 1.5 min in 10s cycles	Release ability	K = 1.8h^{-1} (1.5 – 2.1); %R_∞ = 92.0 % (85 – 99)
	Ethyl acetate Rotary evaporation	Permeation capacity (skin retention)	Superficial skin layers, manly in the epidermis

Table S2. Quality Target Product Profiles (QTPPs) for the KT-formulations.

	Quality target product profiles (QTPPs)	Target
**KT-CTS gel**	Dosage form	Semisolid hydrogel. Easy to apply and conserve.
	Route of administration	Dermal. Non-invasive routewhich allows higher drug concentration on the application site avoiding systemic side effects.
	Site of activity	Superficial skin layers. Epidermis.
	Therapeutic effect	Analgesic and anti-inflammatory effect for the post-operative management of condyloma removal.
	Appearance	Homogeneous and transparent to ensure the aesthetic appearance and increase patient compliance.
	Viscosity	Sufficient consistency to not spread to unwanted areas as soon as the patient changes position, but it must fluidize just enough to allow an easy and painless application.
	Stability	No visible sign of instability at the time of preparation and after three months at room temperature (ca. 25°C).
	Release profile	Controlled drug release. A sustained release over time is desirable for the management of post-surgical pain and inflammation.
**KT-NPs**	Dosage form	Colloidal suspension suitable for sprayable application onto warts without the need to contact them.
	Route of administration	Dermal. Non-invasive route which allows higher drug concentration on the application site avoiding systemic side effects.
	Site of activity	Superficial skin layers. Epidermis.
	Therapeutic effect	Analgesic and anti-inflammatory effect for the pre-operative management of condyloma removal.
	Appearance	Homogeneous fluid with no visible suspended particles
	Stability	No visible sign of instability at the time of preparation and after three months at 4ºC storage conditions. pH, Z-awe, PI, ZP and EE% should not be altered.
	Release profile	Controlled drug release. Rapid drug release is desirable for management of pre-surgical pain and inflammation

Table S3. Optimization of the KT-NPs by a Two-level Full Factorial Design for 3 factors in standard order. .

Run	X_1	X_2	X_3	Amount KT (mg)	Amount PLGA (mg)	pH inneraqueousphase
F1	−1	−1	−1	20	90	2.0
F2	1	−1	−1	80	90	2.0
F3	−1	1	−1	20	110	2.0
F4	1	1	−1	80	110	2.0
F5	−1	−1	1	20	90	7.5
F6	1	−1	1	80	90	7.5
F7	−1	1	1	20	110	7.5
F8	1	1	1	80	110	7.5

X_1, X_2 and X_3 stands for the three factors evaluated: amount of KT in the formulation, amount of PLGA in the formulation and pH of the inner aqueous phase, respectively.

Table A4 shows the responses for each formulation. The following characteristics were measured: particle size (Zave), polydispersity index (PI), zeta potential (ZP), encapsulation efficiency (EE%) and the final pH of the external aqueous phase (W_2).

Table S4. Responses from KT-NPs formulated based on the Two-level Full Factorial Design for 3 factors.

	Factors			Responses				
**Formulation**	**Amount KT (mg)**	**Amount PLGA (mg)**	**pH inneraqueousphase**	**Zave (nm)**	**PI**	**ZP (mV)**	**EE (%)**	**Final pH externalaqueousphase**
F1	20	90	2.0	138.2	0.048	−8.35	92.3	4.0
F2	80	90	2.0	108.9	0.061	−6.20	93.9	5.2
F3	20	110	2.0	136.6	0.047	−11.00	80.7	3.6
F4	80	110	2.0	120.2	0.048	−0.27	71.8	4.8
F5	20	90	7.5	121.8	0.049	−12.40	72.4	5.2
F6	80	90	7.5	110.6	0.043	−7.29	65.2	5.1
F7	20	110	7.5	142.4	0.041	−13.60	35.0	4.7
F8	80	110	7.5	113.6	0.024	−5.57	74.5	5.6

Zave = average particle size; PI = polydispersity index; ZP = zeta potential and EE = Encapsulation efficiency.

The amount of KT or PLGA and the pH of the inner aqueous phase (W1) did not impact on the polydispersity index of the formulations.All of them presented PI values below 0.1 indicating that the colloidal suspension of KT-NPs was a monodisperse system with a narrow particle size distribution. The zeta potential is an important tool to predict NPs stability, the greater the values (positive or negative), the higher stability, with a cut off value of ±30 mV [54]. However, in the case of KT-NPs the ZP value was not critical because the KT-NPs were prepared with PVA as the stabilizer agent. The PVA's mechanism of action to prevent particle aggregation relies on steric hindrance [55].

Taking into account the above mentioned, F2 was selected as the optimized formulation. It was selected based on its smaller particle size and having the highest encapsulation efficiency. Additionally, the pH value was close to the skin's and so this was a desirable characteristic for the dermal delivery.
